# Dr. Maynard's Drill Stock

**Published:** 1842-09

**Authors:** 


*Dr. Maynard's Drill Stock.*—We had hoped to have been able to have given a description of this beautiful and ingeniously constructed instrument in the present number of our Journal, but not having been able to obtain a drawing of it, we are prevented from doing so. We are, however, promised an engraving of it, which shall appear in our next. Although we have never had an opportunity of using the instrument, we do not hesitate to pronounce it a valuable one. Every dentist should have it.

